# Prebiotic Supplementation During Pregnancy Modifies the Gut Microbiota and Increases Metabolites in Amniotic Fluid, Driving a Tolerogenic Environment *In Utero*

**DOI:** 10.3389/fimmu.2021.712614

**Published:** 2021-07-14

**Authors:** Carole Brosseau, Amandine Selle, Angeline Duval, Barbara Misme-Aucouturier, Melanie Chesneau, Sophie Brouard, Claire Cherbuy, Véronique Cariou, Gregory Bouchaud, Kyle T. Mincham, Deborah H. Strickland, Sebastien Barbarot, Marie Bodinier

**Affiliations:** ^1^ Institut National de Recherche pour l’Agriculture, l’alimentation et l’Environnement (INRAE) Pays de la Loire, UR1268 BIA, Impasse Thérèse Bertrand-Fontaine, Nantes, France; ^2^ Centre de Recherche en Transplantation et Immunologie UMR1064, INSERM, Université de Nantes, Institut de Transplantation Urologie Néphrologie (ITUN), CHU Nantes, Labex IGO, Nantes, France; ^3^ INRAE Micalis, AgroParisTech, Université Paris-Saclay, Jouy-en-Josas, France; ^4^ StatSC, ONIRIS, INRAE, Nantes, France; ^5^ Telethon Kids Institute, University of Western Australia, Nedlands, WA, Australia; ^6^ Department of Dermatology, CHU Nantes, Nantes, France; ^7^ UMR PhAN, INRAE, Nantes, France

**Keywords:** prebiotic, pregnancy, gut microbiota, immune tolerance, feto-maternal tissues, immune imprinting

## Abstract

The gut microbiota is influenced by environmental factors such as food. Maternal diet during pregnancy modifies the gut microbiota composition and function, leading to the production of specific compounds that are transferred to the fetus and enhance the ontogeny and maturation of the immune system. Prebiotics are fermented by gut bacteria, leading to the release of short-chain fatty acids that can specifically interact with the immune system, inducing a switch toward tolerogenic populations and therefore conferring health benefits. In this study, pregnant BALB/cJRj mice were fed either a control diet or a diet enriched in prebiotics (Galacto-oligosaccharides/Inulin). We hypothesized that galacto-oligosaccharides/inulin supplementation during gestation could modify the maternal microbiota, favoring healthy immune imprinting in the fetus. Galacto-oligosaccharides/inulin supplementation during gestation increases the abundance of *Bacteroidetes* and decreases that of *Firmicutes* in the gut microbiota, leading to increased production of fecal acetate, which was found for the first time in amniotic fluid. Prebiotic supplementation increased the abundance of regulatory B and T cells in gestational tissues and in the fetus. Interestingly, these regulatory cells remained later in life. In conclusion, prebiotic supplementation during pregnancy leads to the transmission of specific microbial and immune factors from mother to child, allowing the establishment of tolerogenic immune imprinting in the fetus that may be beneficial for infant health outcomes.

## Introduction

Prenatal life is the first period in which the maternal environment can influence the immune system (IS) of the fetus *in utero via* immune factors, bacteria and bacterial metabolites transferred through the cord blood, placenta and amniotic fluid (1–3). These bacteria, their DNA or their metabolites, could trigger immune responses in the fetus and would therefore program the infant’s immune development during fetal life. These observations have led to the notion of “early life imprinting” of the IS (4). The microbiota is influenced by environmental factors, including food (5). Therefore, several studies have investigated the effects of maternal nutrition or food supplementation on maternal gut microbiota modulation during pregnancy and its association with childhood diseases or the beneficial effect of food intake on disease prevention (The concept of the developmental origins of health and disease, DOHaD) (5). For example, maternal intake of omega-3 or polyunsaturated fatty acids is associated with protection against allergic outcomes in children (6). In this context, antioxidants, folates, vitamin D and probiotics have been tested (7). How food supplementation during pregnancy increases diversification of the intestinal microbiota by promoting the emergence of bacteria that benefit the host is currently one of the most widely investigated questions in this field. However, much remains to understood concerning the contribution of the maternal microbiota to immune cell imprinting and therefore susceptibility to infections and immunopathology later in life.

Prebiotics are nondigestible foods composed of linked sugars such as oligosaccharides and short-chain polysaccharides. They have been described as “substrates selectively used by microorganisms of the host conferring benefits for his health” (8). The benefits of prebiotics are not limited to the gut; they can act systemically 1) as a fermentable substrate for some specific commensal host bacteria, leading to the release of short-chain fatty acids (SCFAs), or 2) by exerting direct effects on several compartments and specifically on different types of cells, such as epithelial and immune cells (9). Consumption of prebiotics such as inulin or galacto-oligosaccharides (GOS) increases the relative abundance of the major phyla *Firmicutes, Bacteroidetes, Actinobacteria* and *Proteobacteria* (10). Fermentation of prebiotics by specific taxa leads to the release of SCFAs, mainly acetate, butyrate and propionate, in the gut intestinal tract, and these SCFAs can be used by the microbiota for their own metabolism or released into the bloodstream. In the blood, SCFAs can specifically interact with different cells, such as intestinal epithelial cells or innate/adaptive immune cells, to modify various cellular processes, such as gene expression, differentiation, proliferation and apoptosis (11). For example, SCFAs can modify the hematopoiesis of dendritic cell (DC) precursors in the bone marrow (12). In the intestine, the consumption of SCFAs increases the frequency of tolerogenic cDCs, inducing T cell differentiation into Tregs (13).

During pregnancy, the maternal immune system (IS) must maintain tolerance to the fetal allograft while preserving innate and adaptive immune mechanisms for protection against microbial infections (14). The presence of the placenta and the decidua, which are tightly regulated by immune organs, modifies maternal immunity and physiology to sustain pregnancy and prevent the embryo from being attacked by maternal immune cells (15, 16). One mechanism that plays a crucial role in the maintenance of a successful pregnancy is the switch from a T helper (Th) Th1 cytokine profile to a Th2 profile. Expansion of Tregs in the placenta and decidua of pregnant women, combined with interactions with cDCs, suppresses maternal Th1 activity (17). Concerning B lymphocytes, it was shown that during pregnancy, the percentages of transitional B cells and regulatory B cells (Bregs) were lowered in the peripheral blood (18). This reduction was suggested to be due to the migration of these cells to the maternal-fetal interface, although this was never confirmed. Fetal life is a critical period during which the IS starts to develop in the embryo at an estimated gestational age of approximately 5 weeks (equivalent to 11 days of gestation in mice), with the emergence of hematopoietic stem cells (HSCs) (19). Waves of immune cell ontogeny occur in the yolk sac, liver and bone marrow. This leads to the timely production of the main immune cell populations including macrophages, B and T cells (20). Interestingly, it was demonstrated that mother food intake during pregnancy have a direct impact on the ontogeny of these immune cells and may exert an influence on the emerging immune system *via* epigenetic mechanisms (21).

At the start of pregnancy and during the first trimester, the microbial diversity in the gut appears to be similar to that of nonpregnant women. However, a substantial shift in phylogenetic composition and structure occurs over the course of pregnancy (22). By the third trimester, gut microbiota changes are associated with vastly expanded diversity, an overall increase in the abundance of *Proteobacteria* and *Actinobacteria*, and reduced richness (23). For instance, *Faecalibacterium* is less abundant on average during the third trimester. The biological purpose of these shifts may be to contribute to the ability of the mother to adapt to pregnancy and facilitate optimal fetal growth and development. In a cohort of paired maternal and fetal serum, Hu et al., found a correlation between the acetate levels in cord blood and maternal blood. This correlation suggests that maternal SCFAs are likely to cross the placenta and therefore influence fetal SCFA levels by modifying fetal immune function (24). Interestingly, Thorburn et al. showed that the maternal microbiota imprints the fetal lung, with increased generation of Tregs in adult offspring, reducing the severity of house dust mite-induced allergies (25). These studies, among others, demonstrate the existence of a window of opportunity during pregnancy, in which the fetus might be exposed to products or metabolites of the maternal microbiota that facilitate development of a balanced immune system. It has already been established that particular bacterial strains promote healthy imprinting, such as fermenters of dietary fibers that generate SCFAs; these bacteria belong to the *Bifidobacterium*, *Bacteroidetes* and *Lactobacillus* families (26). How nutrition modulate the maternal microbiota during pregnancy and shape the neonatal immune system was reviewed by Macpherson and colleagues (5).

In this study, pregnant mice were fed either a control diet or a diet enriched in GOS/Inulin. We hypothesized that GOS/inulin supplementation during gestation could modify the maternal microbiota toward higher SCFA production, leading to a tolerogenic environment *in utero* and favoring healthy immune imprinting in the fetus.

## Material and Methods

### Animal Model

Six-week-old BALB/cJRj mice were purchased from Janvier labs (Le Genest-Saint-Isle, France) and housed at constant temperature (20°C) and humidity (40-60%) in a ventilated cage system under a 12:12 h light/dark cycle in the animal facility of INRAE BIA in Nantes. The protocol was approved by the Ethics Committee on Animal Experimentation of the Pays de la Loire region (CEEA.2011.52; 128; accreditation number: 14035).

### Time-Mated Pregnancies

Female BALB/cJRj mice 7-17 weeks of age were time-mated with male BALB/cJRj studs 7-17 weeks of age. Forty-eight hours before mating, straw from the male cage was placed in the female cages to induce mouse estrus to optimize the chances of fertilization (27). Male studs were housed individually with 1-2 females overnight. The following morning was designated GD1.

### Prebiotic Supplementation

Mice were fed either a standard diet or a diet supplemented with 4% galacto-oligosaccharides (FrieslandCampina, Netherlands) and inulin (Beneo Orafti, Belgium) at a 9:1 ratio (Safe, France) during mating and pregnancy as previously used (28–30). The composition of the food was developed to provide all the nutrients necessary during mouse gestation.

### Tissue Collection

Pregnant BALB/cJRj mice were sacrificed at GD18. Amniotic fluid was collected and frozen at -80°C. Both horns of the uterus, decidua and placenta were collected and placed into cold phosphate-buffered saline (PBS). Fetuses were sacrificed by decapitation. Blood was collected, and the fetal hind legs (cleaned of excess tissue) and intestines were removed and placed into cold PBS. Finally, the spleen and femur from the mother were also collected and placed into cold PBS.

### Preparation of Single-Cell Suspensions

The uterus, decidua, placenta and fetal hind legs were prepared by mincing with a scalpel followed by enzymatic digestion, as previously detailed (31). Briefly, minced tissue was resuspended in 10 ml GKN buffer (pH 7.2: 8 g NaCl, 0.4 g KCl, 3.56 g Na_2_HPO_4_.12H_2_O, 0.78 g NaH_2_PO_4_.2H_2_O, and 2 g D-glucose in 1 L H2O) + 10% fetal calf serum (FCS) (Eurobio Scientific; France) with collagenase IV (Worthington Biochemical Corp.) and DNase (Sigma-Aldrich, France) at 37°C under gentle agitation for 60 minutes. The digested cells were filtered, centrifuged and resuspended in cold PBS for total cell counts. The maternal spleen and fetal intestine were mashed, filtered and resuspended in cold PBS for total cell counts. Finally, bone marrow cells from the mother femur were collected by flushing the bone with a syringe, filtered and resuspended in cold PBS for total cell counts.

### Flow Cytometry

A panel of monoclonal antibodies was developed to enable phenotypic characterization of B and T lymphocyte subpopulations: CD3-FITC, CD4-APC, CD25-BV421, FoxP3-PE, CD19-PeCy7, CD9-BB700 (BD Bioscience, France), CD24-BV510, and CD38-APC-Cy7 (Sony Biotechnology, UK); hematopoietic stem and progenitor cells: CD34-BV421 (Biolegend, France), cKit-APC-H7, CD135-PE, lineage-FITC, Sca-1-BV510, CD16/32-PerCPCy5.5, CD127-APC (BD Bioscience), and TLR-4-PeCy7 (Biolegend); and dendritic cells: CD11c-PeCy7 (eBioscience, France), CMH-II-FITC, Siglec-H-BV510, CD11b-APC-H7, and CD103-PerCP5. For intracellular staining, cells were fixed and permeabilized using a Cytofix/Cytoperm kit (BD Biosciences). For *in vitro* human B cell differentiation experiments, cells were stained with Fixable Viability Dye eFluor 450 to identify dead cells, followed by staining with CD25-BV605 (Biolegend), CD19-BUV395, CD9-FITC, CD27-BUV737, CD38-BV711 and intracellular IL-10-PE (BD Bioscience). Cells were analyzed on a Canto II flow cytometer (BD Biosciences). Data were acquired using Diva 8.0 software and analyzed with FlowJo X (TreeStar, Williamson Way, Ashland, USA). Fluorescence minus one staining controls were used for all panels, and dead cells were removed using viability staining.

### Human B Cell Activation *In Vitro*

Fresh human peripheral blood mononuclear cells (PBMCs) were isolated from the whole blood of healthy donors using Ficoll gradient centrifugation. B cells were negatively selected from human PBMCs by magnetic separation using a human B cell isolation kit II (Miltenyi Biotec, France). B cells were cultured at 10^6^ cells/ml in RPMI 1640 medium (Thermo Fisher, France) supplemented with 10% FCS, L-glutamine, and penicillin/streptomycin. B cells were activated in the presence of F(ab’)2 anti-BCR Abs (5 µg/ml) (Jackson ImmunoResearch, France), CpG ODN 2006 (2 µg/ml) (Invivogen, France), and CD40L (100 ng/ml) (R&D Systems, France). GOS/inulin (1 mg/mL), butyrate (0.1 mM), propionate (1 mM) and acetate (10 mM) (Sigma-Aldrich) were added to the B cell culture for 3 days. Four hours before cell staining, brefeldin A (Thermo Fisher) was added at 5 µg/ml.

### Analysis of the Fecal Microbiota Community by 16S rRNA Gene, 16S rDNA Gene Sequencing and Statistical Analysis

Fecal microbiota, genes survey and sequences analysis was performed as described by Cherbuy and colleagues (32). Total bacterial DNA was extracted from the collected samples using the QIAamp power faecal DNA kit (Qiagen), and DNA quantity was determined using a TECAN Fluorometer (Qubit^®^ dsDNA HS Assay Kit, Molecular Probes). The V3-V4 region of the 16S rRNA gene was amplified by PCR using the following primers: a forward 43-nuclotide fusion primer 5′CTT TCC CTA CAC GAC GCT CTT CCG ATC TAC GGR AGG CAG CAG3′ consisting of the 28-nt illumina adapter (bold font) and the 14-nt broad range bacterial primer 343F and a reverse 47-nuclotide fusion 5′GGA GTT CAG ACG TGT GCT CTT CCG ATC TTA CCA GGG TAT CTA ATC CT3′ consisting of the 28-nt illumina adapter (bold font) and the 19-nt broad range bacterial primer 784R. The PCR reactions were performed using 10 ng of DNA, 0.5 µM primers, 0.2 mM dNTP, and 0.5 U of the DNA-free Taq-polymerase, MolTaq 16S DNA Polymerase (Molzym). The amplifications were carried out using the following profile: 1 cycle at 94°C for 60 s, followed by 30 cycles at 94°C for 60 s, 65°C for 60 s, 72°C for 60 s, and finishing with a step at 72°C for 10 min. The PCR reactions were sent to the @Bridge platform (INRAE, Jouy-en-Josas) for sequencing using Illumina Miseq technology. Single multiplexing was performed using home-made 6 bp index, which were added to R784 during a second PCR with 12 cycles using forward primer (AATGATACGGCGACCACCGAGATCTACACTCTTTCCCTACACGAC) and reverse primer (CAAGCAGAAGACGGCATACGAGAT-index-GTGACTGGAGTTCAGACGTGT). The resulting PCR products were purified and loaded onto the Illumina MiSeq cartridge according to the manufacturer instructions. The quality of the run was checked internally using PhiX, and then, sequences were assigned to its sample with the help of the previously integrated index. Sequences were assembled and processed using FROGS pipeline (Find Rapidly OTU with Galaxy Solution) to obtain OTUs and their respective taxonomic assignment thanks to Galaxy instance (https://migale.inra.fr/galaxy). The successive steps involved de-noising and clustering of the sequences into OTUs using SWARM; chimera removal using *vs.*EARCh; taxonomic affiliation for each OTU using both RDPClassifier and NCBI Blast+ on Silva SSU 138 40. Statistical analyses were performed using R software, version 3.2.3 (R Core Team, 2020). β-diversity (Unifrac dissimilarity). α-diversity measurements and analysis of the differences in OTUs between samples were performed using the R package Phyloseq. Differences in the microbial communities between the CTL and the PB groups were evaluated using constrained analysis of principal coordinates and permutational multivariate analysis of variance (PERMANOVA). The differential abundances of bacterial taxa were tested with the DESeq2 R package, which is based on negative binomial generalized linear models 43. False discovery rate corrected P values below 0.05 were considered significant.

### SCFA Analysis of Fecal Samples

The acetate, propionate and butyrate fecal concentrations were determined by gas chromatography as described by Cherbuy et al. (32). SCFA (acetate, propionate and butyrate) content was determined by gas chromatography. The faecal samples were extracted with water (wt g/vol), centrifuged at 17,000 x g for 10 min, and the supernatant collected. The proteins were precipitated using a phosphotungstic acid saturated solution. Supernatant was analyzed using a gas chromatograph (GC 7890, Agilent Technologies, France). All samples were analyzed in duplicate. The data was collected and peaks integrated using Agilent Technologies software.

### NMR-Based Metabolic Fingerprints of Amniotic Fluid

**Figure 1:**
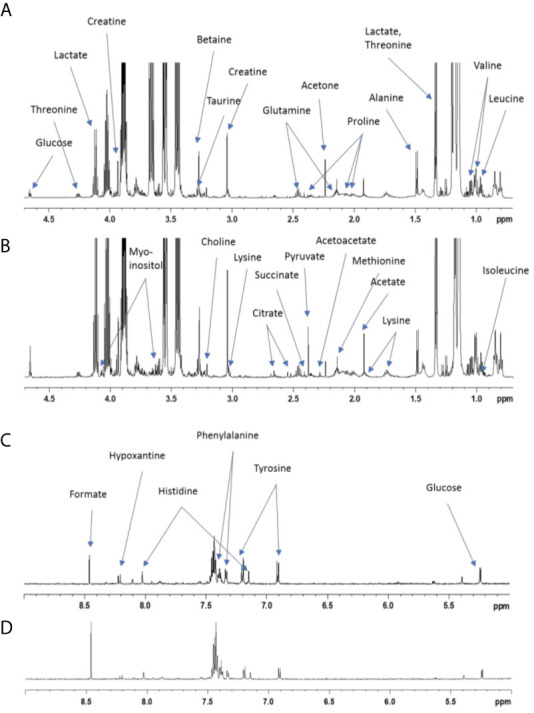
NMR spectra of amniotic fluid collected from dams supplemented or not with GOS/inulin. The 600 MHz NMR spectra of liquid amniotic samples **(A)** from the control group sample and **(B)** from the prebiotic group sample; zoom between 4.7 and 0.7 ppm. The 600 MHz NMR spectra of liquid amniotic samples **(C)** from the control group sample and **(D)** from the prebiotic group sample; zoom between 9 and 5 ppm.

1H NMR spectra were obtained at 300 K on a Bruker Avance III HD 600 MHz NMR spectrometer (Bruker Biospin, Rheinstetten, Germany), operating at 600.13 MHz for the 1H resonance frequency using an inverse detection 5 mm 1H-13C-15N-31P cryoprobe attached to a Cryoplatform. “Tuning” and “matching” of the probe, locking, shim tuning, pulsing (90°) and gain computation are automatically performed for each sample. 1H NMR spectra were acquired using the 1D CPMG experiment with presaturation for water and macromolecule suppression (cpmgpr1d), with a spin-echo delay of 240 ms. A total of 128 transients were collected into 64k data points using a spectral width of 12 ppm, a relaxation delay of 5 s and an acquisition time of 4.55 s. Prior to Fourier transform, an exponential line broadening function of 0.3 Hz was applied to the FID. All NMR spectra were phase- and baseline-corrected and referenced to the chemical shift of TSP (0 ppm) using Topspin (V3.2, Bruker Biospin, Germany). The NMR spectra of amniotic fluid were then divided into fixed-size buckets (0.01 ppm) between 9 and 0.5 ppm using AMIX software (v3.9.15, Bruker) (**Figure 1**), and the area under the curve was calculated for each bucket. The regions including residual water (5.2-4.4 ppm) and ethanol signals (3.70-3.60 and 1.32-1.06 ppm) were removed. Integrations were normalized according to the total intensity. A PCA of metabolic fingerprints followed by a PLS-DA were then performed to evaluate discrimination between supplementation levels. These multivariate methods were described by Cabaton (33). NMR buckets with VIP > 1.0 were selected as discriminants. Finally, a nonparametric univariate Wilcoxon test was performed on metabolic features from multivariate analysis. The false discovery rate (FDR) was applied to take into account multiple testing and avoid false positives. Statistical analyses were conducted with Simca software (V15; Umetrics AB, Umea, Sweden) and R [in house scripts and the ropls package (34)].

### Statistics

Results in the **Table 1** were expressed as mean ± standard deviation and the results in the figures were represented in Box and Whisker Plot showing the intervals and the median. Comparisons of experimental values between the two groups were analyzed using the Wilcoxon and the Mann–Whitney U-test. All statistical analyses were performed in GraphPad Prism v7, La Jolla, USA.

**Table 1:** Effects of GOS/inulin supplementation during pregnancy on gestational outcomes.

	Food intake (g/w)	Mating efficiency (%)	Maternal weight (g)	Fetus weight (mg)	Litter size	Number of resorption
**Control**	18.7 ± 0.1	24 ± 6	32.6 ± 0.8	780 ± 17	7.2 ± 0.8	1 ± 0.2
**Prebiotics**	18.5 ± 0.2	30 ± 6	35.3 ± 0.9	758 ± 21	7.7 ± 0.5	1.6 ± 0.3

Assessment of food intake per week, mating efficiency percentage, maternal weight and fetal weight at 18 days of gestation, litter size and the number of fetuses resorbed for the control diet and GOS/inulin diet groups (mean ± standard deviation).

## Results

### GOS/Inulin Supplementation During Pregnancy Has No Effect on Gestational Outcomes

First, the addition of GOS/inulin to the diet had no significant effect on the amount of food intake by pregnant mice (18.7 ± 0.1 g/w *vs.* 18.5 ± 0.2 g/w for mice fed a control diet (n=15) *vs.* those supplemented with prebiotics (n=12), respectively, p=0.4) (**Table 1**). Mating efficiency, which corresponds to the percentage of pregnant mice among mated mice, was also similar between the two groups (24% ± 6 *vs.* 30% ± 6 for mice fed a control diet *vs.* mice supplemented with prebiotics, respectively, p=0.4). At 18 days of gestation (GD18), prebiotic supplementation had no effects on maternal weight (32.6 ± 0.8 g *vs.* 35.3 ± 0.9 g for mice fed a control diet *vs.* mice supplemented with prebiotics, respectively, p=0.3) or fetal weight (780 ± 17 mg *vs.* 758 ± 21 mg for mice fed a control diet *vs.* mice supplemented with prebiotics, respectively, p=0.5). The average pup number per litter did not differ significantly between the two groups (7.2 ± 0.8 *vs.* 7.7 ± 0.5 for mice fed a control diet *vs.* mice supplemented with prebiotics, respectively, p=0.38), nor did the number of resorptions (1 ± 0.2 *vs.* 1.6 ± 0.3 for mice fed a control diet *vs.* mice supplemented with prebiotics, respectively, p=0.5). In conclusion, we found no significant effect of prebiotic supplementation during pregnancy on gestational outcomes.

### The Composition and Metabolism of the Maternal Gut Microbiota Are Modified During GOS/Inulin Supplementation

**Figure 2:**
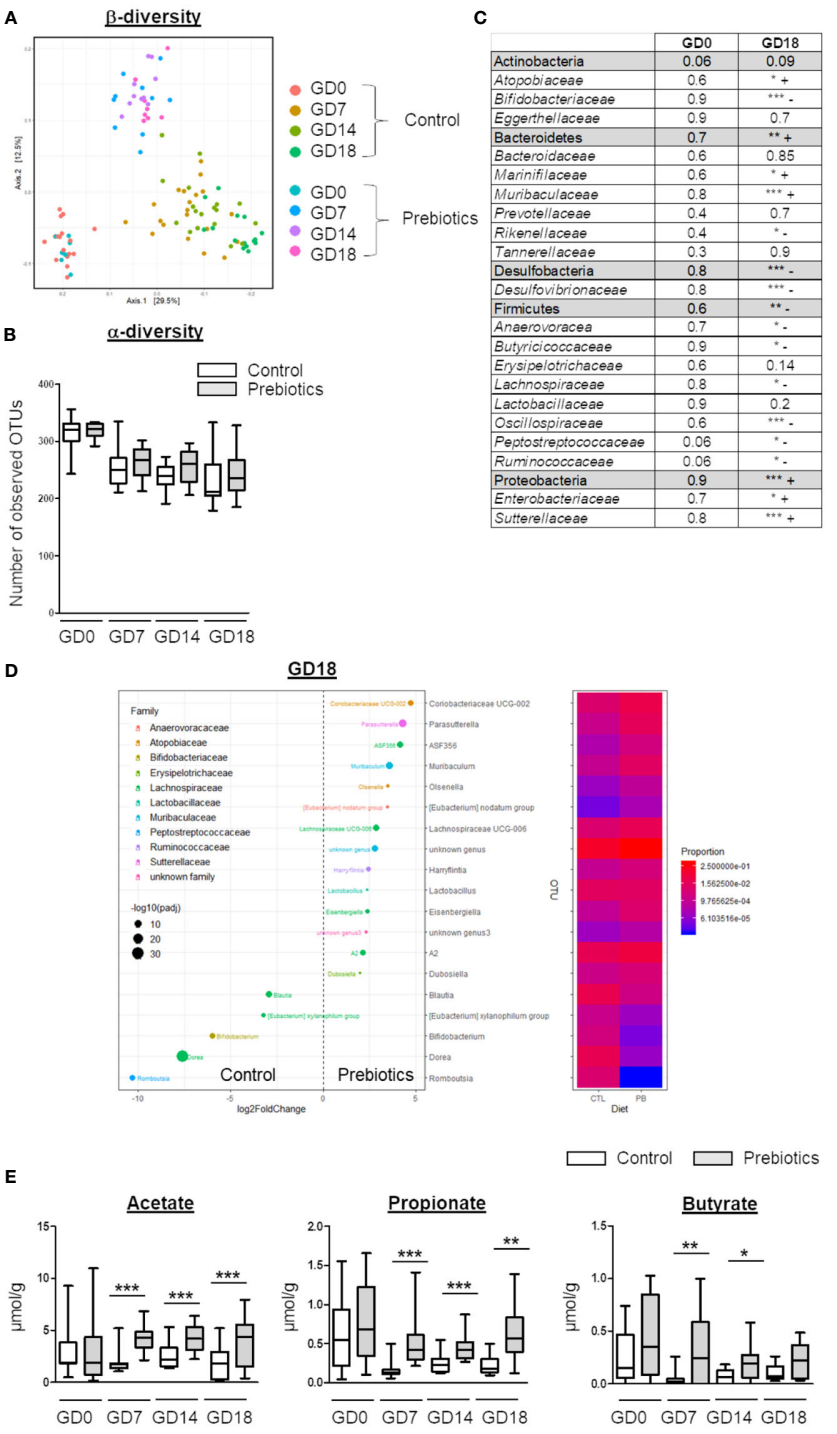
Effects of GOS/inulin supplementation during pregnancy on the fecal microbiota composition and SCFA levels. **(A)** Analysis of β-diversity by unweighted UniFrac-based PCoA of stools from dams fed a control or prebiotic diet before gestation (GD0) and at 7, 14 and 18 gestational days (GD7, GD14 and GD18, respectively). **(B)** α-Diversity of stools from dams fed a control (white) or prebiotic diet (gray) on GD0, GD7, GD14 and GD18. **(C)** Table summarizing the relative abundance of phyla and families in the fecal microbiota. “+” indicates an increase and “-” indicates a decrease in the prebiotic group compared to the control group (*=p<0.05, ** = p<0.01, *** = p<0.001). **(D)** Graphical representation and heatmap of sex variance in stools from dams fed a control or prebiotic diet at GD18. **(E)** Evaluation of SCFA (acetate, propionate, and butyrate) levels in stools from dams fed a control (white) or prebiotic diet (gray) on GD0, GD7, GD14 and GD18 (** = p<0.01, *** = p<0.001).

To evaluate the impact of GOS/inulin supplementation on the microbiota of pregnant mice, stools were collected at gestational day 0 (GD0) before GOS/inulin supplementation and at GD7, GD14 and GD18. Stools were analyzed by 16S rRNA sequencing to evaluate microbial diversity (α and β) and by gas chromatography to measure SCFA (acetate, propionate and butyrate) levels. As expected, no difference in gut microbiota composition was seen before prebiotic supplementation (**Figure 2A, B**). At 7 days of gestation and thereafter, we observed a significant difference in β-diversity between the control and the prebiotic-supplemented mothers, with samples clustered according to diet (p<0.001 ***) (**Figure 2A**), while the α-diversity remained similar over time (**Figure 2B**).

On GD18, the relative abundance of 100 operational taxonomic units (OTUs) significantly increased among the microbiota of prebiotic-supplemented dams compared to control dams, while the abundance of 79 OTUs decreased (listed in **Supplementary Table 1** and summarized **Figure 2C**). In particular, the relative abundance of Bacteroidetes phyla was increased by prebiotic consumption, together with an increased relative abundance of OTUs from the *Muribaculaceae* family (**Figure 2D**). The relative abundance of Desulfobacteria and Firmicutes was decreased, mainly associated with a decrease in OTUs from the *Oscillospiraceae* family and reshaping of the *Lachnospiraceae* family. Finally, the abundance of the Proteobacteria phylum was increased due to an overrepresentation of the *Sutterellaceae* family in the supplemented group compared to the control group. Modification of the β-diversity in prebiotic-supplemented mothers was associated with a significant increase in acetate, propionate and butyrate levels from GD7 (acetate 1.8 ± 0.2 µmol/g of feces *vs.* 4.2 ± 0.3 µmol/g, p<0.001; propionate 0.15 ± 0.02 µmol/g of feces *vs.* 0.53 ± 0.08 µmol/g, p<0.001; butyrate 0.04 ± 0.01 µmol/g of feces *vs.* 0.31 ± 0.08 µmol/g, p<0.001, for the control and the prebiotic groups, respectively) (**Figure 2E**). On GD18, acetate and propionate levels were still significantly higher in the stools of prebiotic-supplemented dams than in the stools of control dams (acetate 1.8 ± 0.3 µmol/g of feces *vs.* 3.9 ± 0.5 µmol/g, p<0.001; propionate 0.2 ± 0.02 µmol/g of feces *vs.* 0.53 ± 0.09 µmol/g, p<0.01). In contrast, the butyrate level did not differ significantly between the two groups. In conclusion, GOS/inulin supplementation during gestation modifies the gut microbiota by increasing *Muribaculaceae* abundance and by reshaping *Lachnospiraceae*, leading to an increase in SCFA concentrations in the stool.

### GOS/Inulin Supplementation Increases Acetate in the Amniotic Fluid

**Figure 3:**
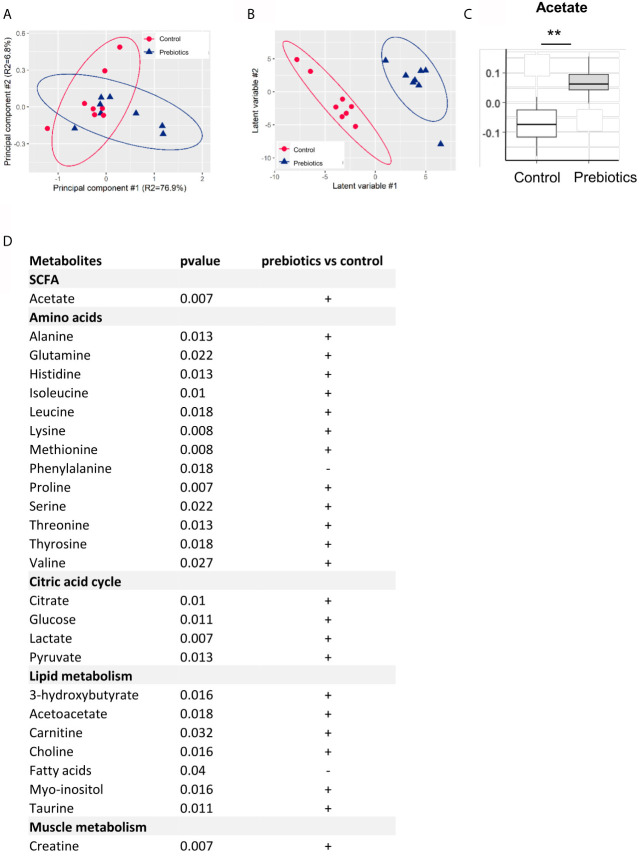
Effects of GOS/inulin supplementation on the concentrations of amniotic fluid metabolites. **(A)** Two-dimensional PCA score plot of 1H NMR integrated spectra of amniotic fluid samples (circle: control, n = 8; triangle: supplementation, n = 8). **(B)** Two-dimensional PLS-DA score plot of OSC-filtered and Pareto-scalde 1H NMR integrated spectra of amniotic fluid samples (circle: control, n = 8; triangle: supplementation, n = 8). **(C)** Boxplot of discriminant (VIP ≥ 1.0) and significant (FDR-corrected p-value ≤ 0.05) features: comparison of the median concentrations of butyrate and acetate between the control (white) and prebiotic-supplemented (gray) groups. **(D)** Table of discriminant metabolites in the amniotic fluid of dams fed a control or prebiotic diet. **p < 0.01.

Then, we investigated the effect of GOS/inulin supplementation on the concentrations of metabolites in the amniotic fluid. Amniotic fluid was collected on GD18 and analyzed by nuclear magnetic resonance (NMR). Principal component analysis (PCA) showed no discrimination between samples (**Figure 3A**). Orthogonal signal filtering was applied to remove orthogonal variability not linked to prebiotic supplementation. Partial least squares discriminant analysis (PLS-DA) showed a clear separation between the control and prebiotic-supplemented dams (**Figure 3B**). A total of 170 features were selected on the basis of the variable importance in the projection index (VIP>1) together with a Wilcoxon nonparametric test. The boxplot of acetate levels showed a significantly higher level in the amniotic fluid of prebiotic-supplemented dams than in that of control dams (p=0.007) (**Figure 3C**). Moreover, it appeared that the concentrations of 23 other metabolites were higher in the amniotic fluid of prebiotic-supplemented dams than in that of control dams. These metabolites belong to the amino acid, citric acid cycle, lipid metabolism and muscle metabolism categories (**Figure 3D**). In conclusion, GOS/inulin supplementation during gestation increased the level of acetate in the amniotic fluid and the levels of other metabolites involved in lipid metabolism and energy production.

### GOS/Inulin Supplementation During Pregnancy Has No Effect on Hematopoietic Stem and Progenitor Cell Abundance in Bone Marrow From Dams and Fetuses

**Figure 4:**
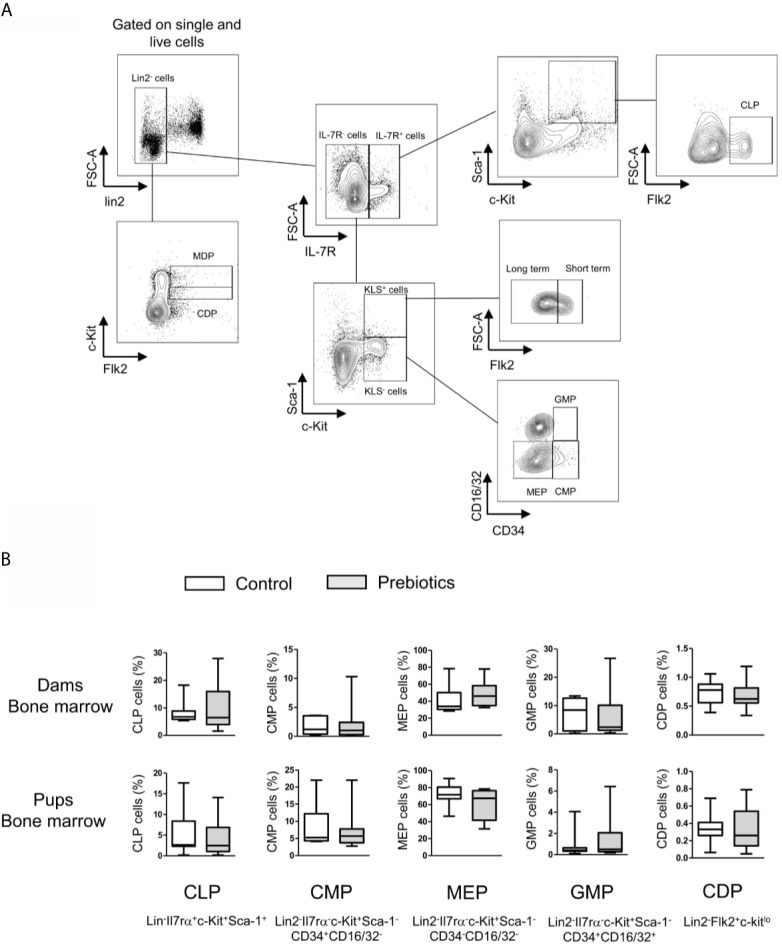
Effects of GOS/inulin supplementation during pregnancy on hematopoietic stem and progenitor cell frequency in bone marrow from dams and fetuses. **(A)** Gating strategy used after immunostaining to evaluate all HSC and progenitor cell subsets. **(B)** Assessment of common lymphoid progenitor (CLP), common myeloid progenitor (CMP), megakaryocyte-erythrocyte progenitor (MEP), granulocyte-macrophage progenitor (GMP) and common DC precursor (DCP) frequencies in the bone marrow of dams and pups fed a control diet (white) or supplemented with GOS/inulin (gray) (n=12).

Thereafter, we analyzed the effect of prebiotics on HSC and progenitor cell abundance in the fetal hind leg and dam femur. More precisely, we determined the frequencies of the following cell subtypes: total HSCs and progenitor cells (c-Kit^+^Lin2^-^Sca-1^+^; KLS phenotype), long-term HSCs (Flk2^-^CD34^-^KLS), short-term HSCs and multipotent progenitors (Flk2^+^CD34^-^KLS), common lymphoid progenitors (Lin^-^Il7rα^+^c-Kit^+^Sca-1^+^ Flk2^+^; CLPs), common myeloid progenitors (Lin2^-^Il7rα^-^c-Kit^+^Sca-1^-^CD34^+^CD16/32^-^; CMPs), megakaryocyte-erythrocyte progenitors (Lin2^-^Il7rα^-^c-Kit^+^Sca-1^-^CD34^-^CD16/32^-^; MEPs), granulocyte-macrophage progenitors (Lin2^-^Il7rα^-^c-Kit^+^Sca-CD34^+^CD16/32^+^; GMPs) (gating strategy is shown in **Figure 4A**) (35). The frequencies of macrophage and DC precursors (Lin2^-^Flk2^+^c-kit^hi^; MDPs) and common DC precursors (Lin2^-^Flk2^+^c-kit^lo^; CDPs) were also estimated (36). The frequencies of all HSCs and progenitor cells were similar between mothers supplemented or not with prebiotics and between fetuses exposed or not to prebiotics *in utero* (**Figure 4B**, frequencies of total KLS cells, MDPs, and long- and short-term KLS cells not shown). In conclusion, prebiotic supplementation during pregnancy has no effect on HSC and progenitor cell frequencies in bone marrow from dams and fetuses.

### GOS/Inulin Supplementation During Pregnancy Has No Effect on DC Abundance in Dams and Their Fetuses

**Figure 5:**
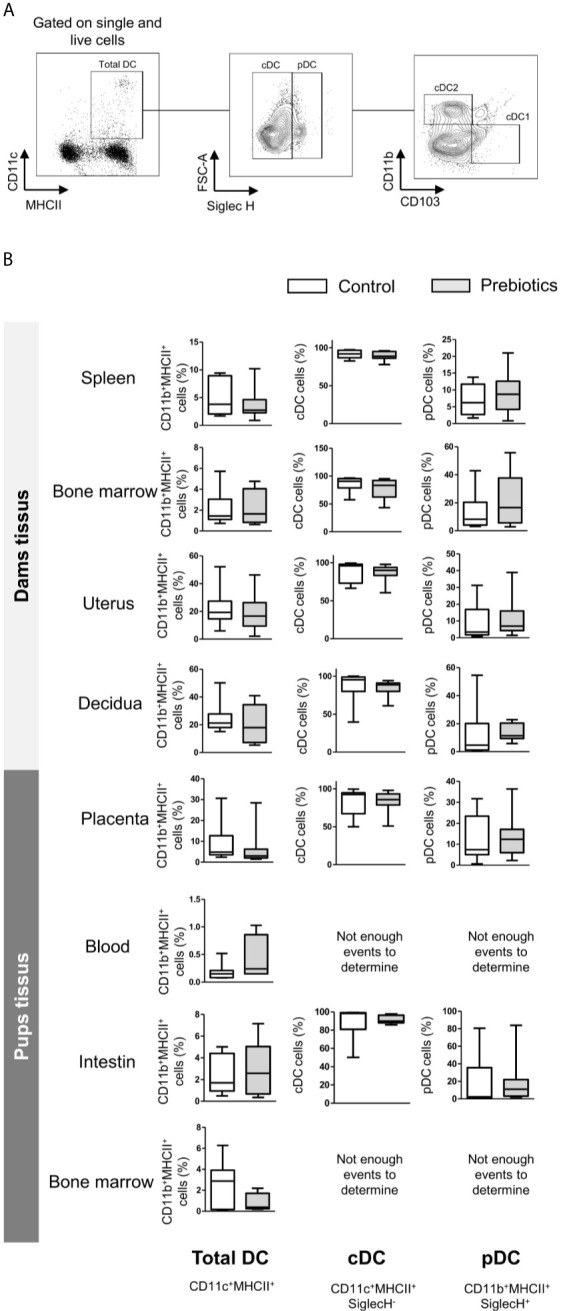
Effects of GOS/inulin supplementation during pregnancy on dendritic cell frequency in dams and fetuses. **(A)** Gating strategy used after immunostaining to evaluate all DC subsets. **(B)** Assessment of total DC, conventional DC (cDC) and plasmacytoid DC (pDC) frequencies in tissues from dams and pups fed a control diet (white) or a diet supplemented with GOS/inulin (gray) (n=9).

We next determined the effects of prebiotics on DC frequencies in the spleen, bone marrow, uterus, decidua, and placenta from mothers supplemented or not with prebiotics. DCs isolated from the blood, intestine and bone marrow of fetuses were also targeted to determine the effects of prebiotic supplementation on fetal DC abundance. The frequencies of total DCs (CD11c^+^CMHII^+^), type 1 DCs (cDC1s; CD11c^+^CMHII^+^SiglecH^-^CD11b^-^CD103^+^), type 2 conventional DCs (cDC2s; CD11c^+^CMHII^+^SiglecH^-^CD11b^+^CD103^-^) and plasmacytoid DCs (pDCs; CD11c^+^CMHII^+^SiglecH^+^) (gating strategy is shown in **Figure 5A**) were evaluated. Prebiotic supplementation had no effects on the frequencies of any DC subpopulations in any tissues studied from dams or fetuses (**Figure 5B**, frequencies of cDC1s and cDC2s not shown).

### GOS/Inulin Supplementation During Pregnancy Increases Regulatory T Cell Frequency in the Placenta and Regulatory B Cell Frequency in Both the Placenta and the Uterus

**Figure 6:**
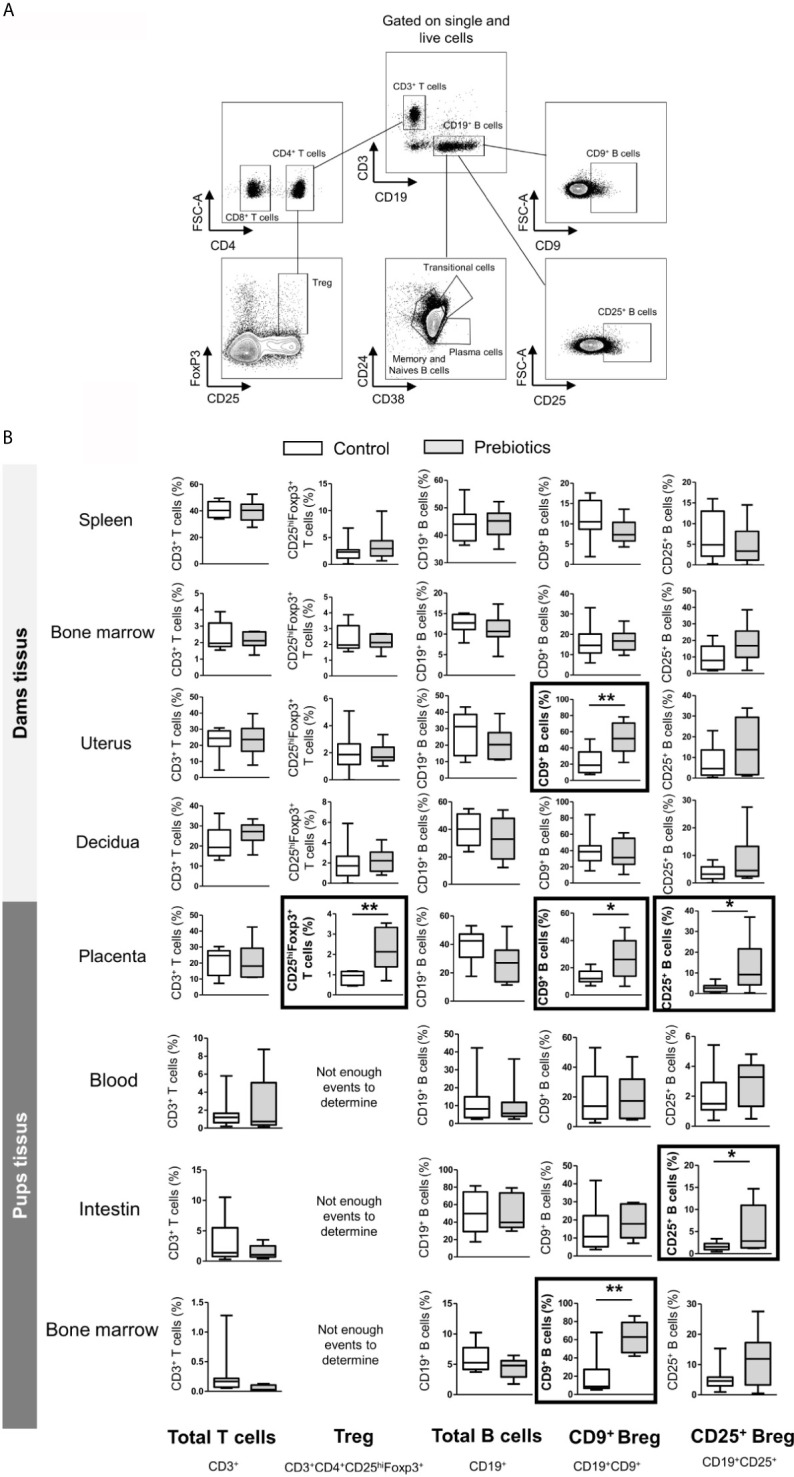
Effects of GOS/inulin supplementation during pregnancy on B and T lymphocyte cell frequency in dams and fetuses. **(A)** Gating strategy used after immunostaining to evaluate all T and B lymphocyte subsets. **(B)** Assessment of total T and B cell, regulatory T cell (Treg) and regulatory B cell (CD9^+^ and CD25^+^ B cell) frequencies in tissues from dams and pups fed a control diet (white) or a diet supplemented with GOS/inulin (gray) (n=9) (* = p<0.05, ** = p<0.01).

We next investigated the potential effect of prebiotic supplementation on the frequencies of T and B cell subpopulations. In the same way described for DCs, T and B cells were isolated from the spleen, bone marrow, uterus, decidua, and placenta from dams supplemented or not with prebiotics. The frequencies of the following subpopulations were estimated: total T cells (CD3^+^), effector T cells (CD3^+^CD4^+^), cytotoxic T cells (CD3^+^CD8^+^), regulatory T cells (CD3^+^CD4^+^CD25^hi^Foxp3^+^), total B cells (CD19^+^), memory B cells (CD19^+^CD27^+^CD38^-^), naive B cells (CD19^+^CD27^-^CD38^-^), transitional B cells (CD19^+^CD24^hi^CD38^hi^) and plasma cells (CD19^+^CD24^-^CD38^+^) (gating strategy is shown in **Figure 6A**). In dam tissues, prebiotic supplementation had no effect on the frequency of proinflammatory T and B lymphocyte subpopulations, such as effector and cytotoxic T cells or conventional B cells (memory, transitional, naive and plasma B cells) (**Figure 6B**, cytotoxic and effector T cells and conventional B cells not shown). Interestingly, we observed that the rate of Tregs was significantly higher in the placenta of supplemented mothers than in the placenta of mothers fed the control diet (0.8% ± 0.1 *vs.* 2.2% ± 0.4 for mice fed a control diet *vs.* mice supplemented with prebiotics, respectively, p ≤ 0.01). In addition, a higher frequency of CD9^+^ Bregs was detected in both the uterus and placenta of supplemented dams compared to control dams (23.3% ± 4.5 *vs.* 52.1% ± 6.3, p ≤ 0.01 in the uterus and 13.3% ± 1.6 *vs.* 27% ± 4.9, p ≤ 0.05 in the placenta for mice fed a control diet or supplemented with prebiotics, respectively). Prebiotic supplementation also significantly increased the frequency of CD25^+^ Bregs in the placenta (2.8% ± 0.7 *vs.* 10.6% ± 4 for mice fed a control diet *vs.* mice supplemented with prebiotics, respectively, p ≤ 0.05). To summarize, prebiotic supplementation during gestation induces increases in Treg frequency in the placenta and Breg frequency in both the placenta and the uterus.

### GOS/Inulin Supplementation During Pregnancy Increases Regulatory B Cell Rate in the Fetus

To determine the effects of prebiotic supplementation during pregnancy on fetal lymphocyte rate, T and B cells were also isolated from the blood, intestine and bone marrow of fetuses. The frequency of total T cells was similar in all tissues between fetuses exposed or not exposed to prebiotics during gestation. Other T cell subpopulations, such as Tregs, could not be estimated due to the small number of events (**Figure 6B**). The frequencies of total B cells and conventional B cells were also similar in all fetal tissues, with or without exposure to prebiotics. However, prebiotic supplementation increased the rate of CD9^+^ Bregs in the fetal bone marrow (16.7% ± 8 *vs.* 57.8% ± 7.8 for fetuses exposed to the control diet or to prebiotics, respectively, p ≤ 0.01) and increased the rate of CD25^+^ Bregs in the fetal intestine (1.7% ± 0.2 *vs.* 4.3% ± 1.5 for fetuses exposed to the control diet or to prebiotics, respectively, p ≤ 0.05). In conclusion, prebiotic supplementation during gestation increases Breg frequency in the bone marrow and intestine of fetuses.

### Placental CD9^+^ and CD25^+^ Breg Cells Induced by Prebiotic Supplementation Produce IL-10

**Figure 7:**
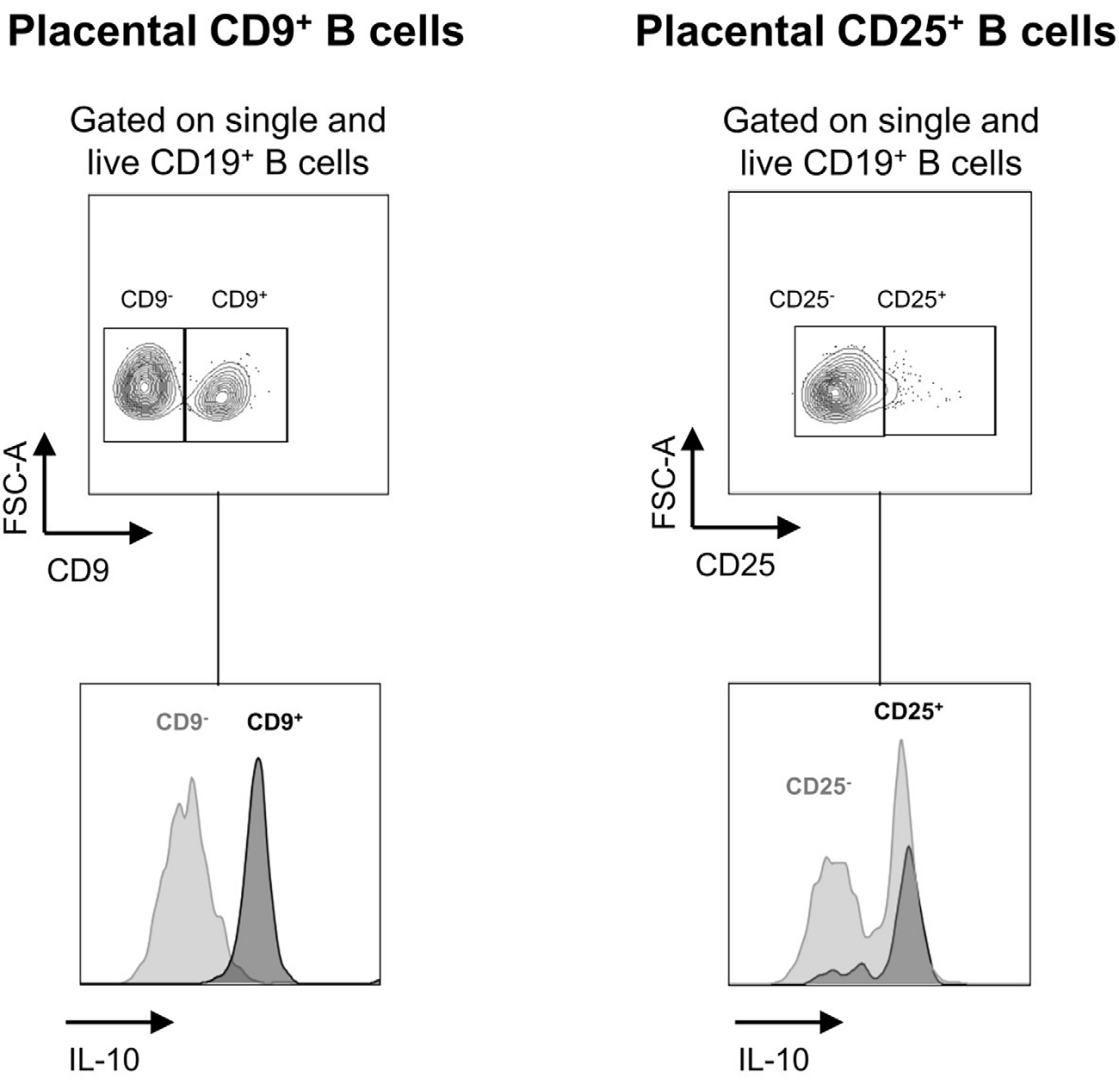
Estimation of IL-10 secretion in CD9^+/-^ and CD25^+/-^ placental B cells induced by GOS/inulin supplementation. Determination of IL-10 secretion by CD9^+^, CD9^-^, CD25^+^ and CD25^-^ B cells in the placenta of dams supplemented with GOS/inulin during gestation by intracellular immunostaining.

To determine whether CD9^+^ Bregs and CD25^+^ Bregs induced by prebiotic exposure in the placenta and fetus have potential regulatory properties, the secretion of IL-10 by these cells was estimated by flow cytometry (**Figure 7**). A total of 92.8% of CD9^+^ Breg cells induced by prebiotics in the placenta secreted IL-10 (4.6% of CD9^-^ B cells secreted IL-10). A total of 81.9% of CD25^+^ Breg cells induced by prebiotics in the placenta secreted IL-10 (51% of CD25^-^ B cells secrete IL-10). IL-10 secretion by Breg cells in the fetus could not be evaluated as these cells died under stimulation. We postulate that they were probably too immature to be activated and/or this stimulation was too strong for them to survive. In conclusion, placental Bregs induced by prebiotic supplementation secrete the anti-inflammatory cytokine IL-10, showing that they probably have regulatory properties.

### The Increase in Fetal Breg Frequency Induced by Prebiotic Supplementation Was Also Observed in Pups at 7 Weeks of Age in Association With Tregs

**Figure 8:**
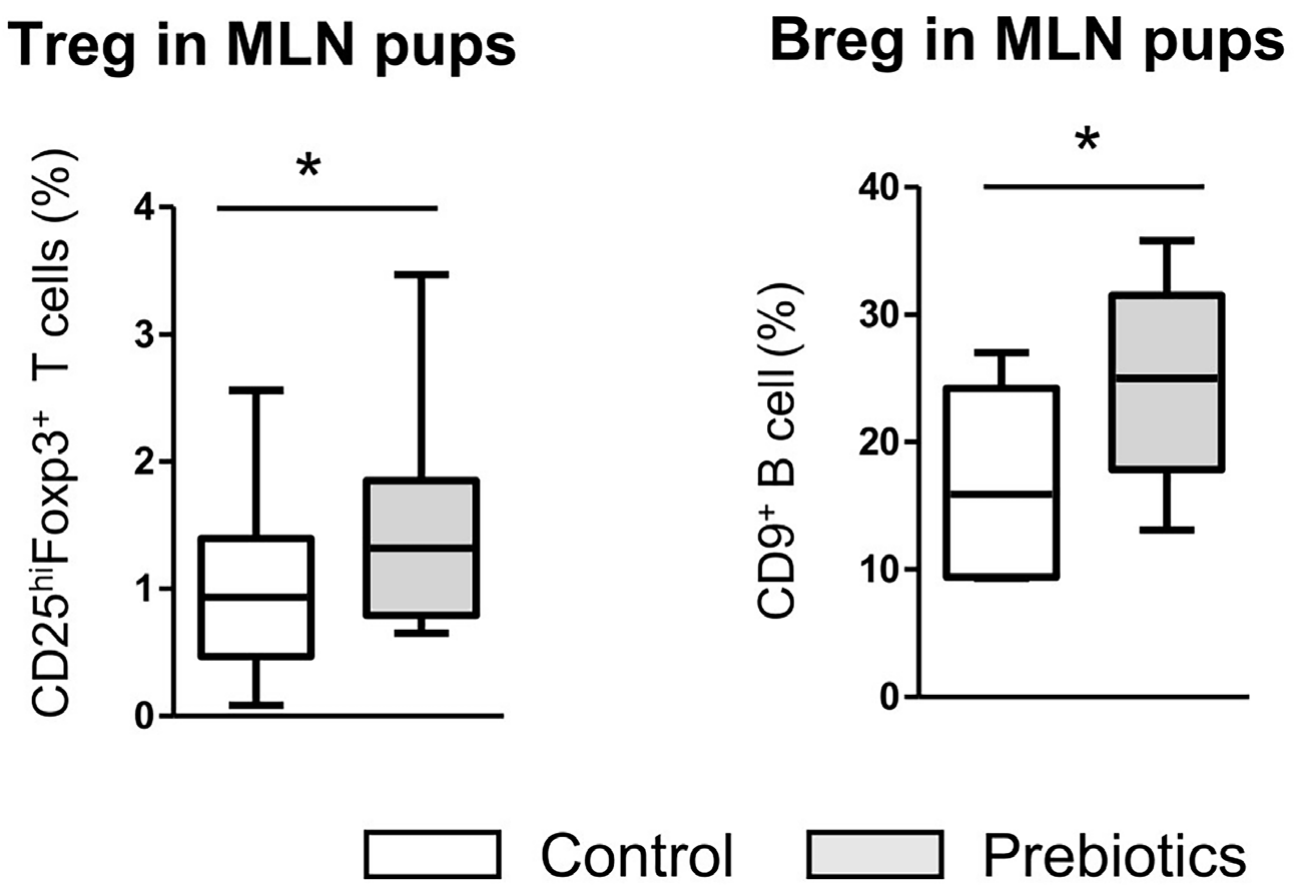
Assessment of Breg and Treg frequencies in the mesenteric lymph nodes of pups at 7 weeks of age. Flow cytometry assessment of CD25^high^FoxP3^+^ Treg cell and CD9^+^ Breg cell frequencies in the mesenteric lymph nodes of pups (7 weeks of age) after GOS/inulin prebiotic exposure during gestation (gray) or the control diet (white) (n=10, * = p<0.05).

We next investigated whether the higher frequency of Bregs observed in fetuses exposed to prebiotics *via* their mothers during gestation was maintained in pups. At 7 weeks of age, the rate of CD9^+^ Bregs was significantly higher in the mesenteric lymph nodes (MLNs) of pups exposed to prebiotics during gestation than in those of nonexposed pups (24.9% ± 2.1 *vs.* 16.8% ± 2.5, respectively, p ≤ 0.05) (**Figure 8**). The level of CD25^+^ Breg cells did not differ significantly between groups (not shown). In contrast to the fetal period, we observed an increase in Tregs in the MLNs of pups exposed to prebiotics during gestation compared to control pups (1.4% ± 0.1 *vs.* 0.9% ± 0.1, respectively, p ≤ 0.05). Thus, prebiotic supplementation during gestation induced higher rate of Bregs that was maintained later in life, which correlated with the establishment of Tregs later in life.

### Acetate but Not Prebiotics Decreases B Cell Differentiation of Proinflammatory CD38^high^CD27^+^ Plasmablasts for the Benefit of IL-10 Regulatory B Cells

**Figure 9:**
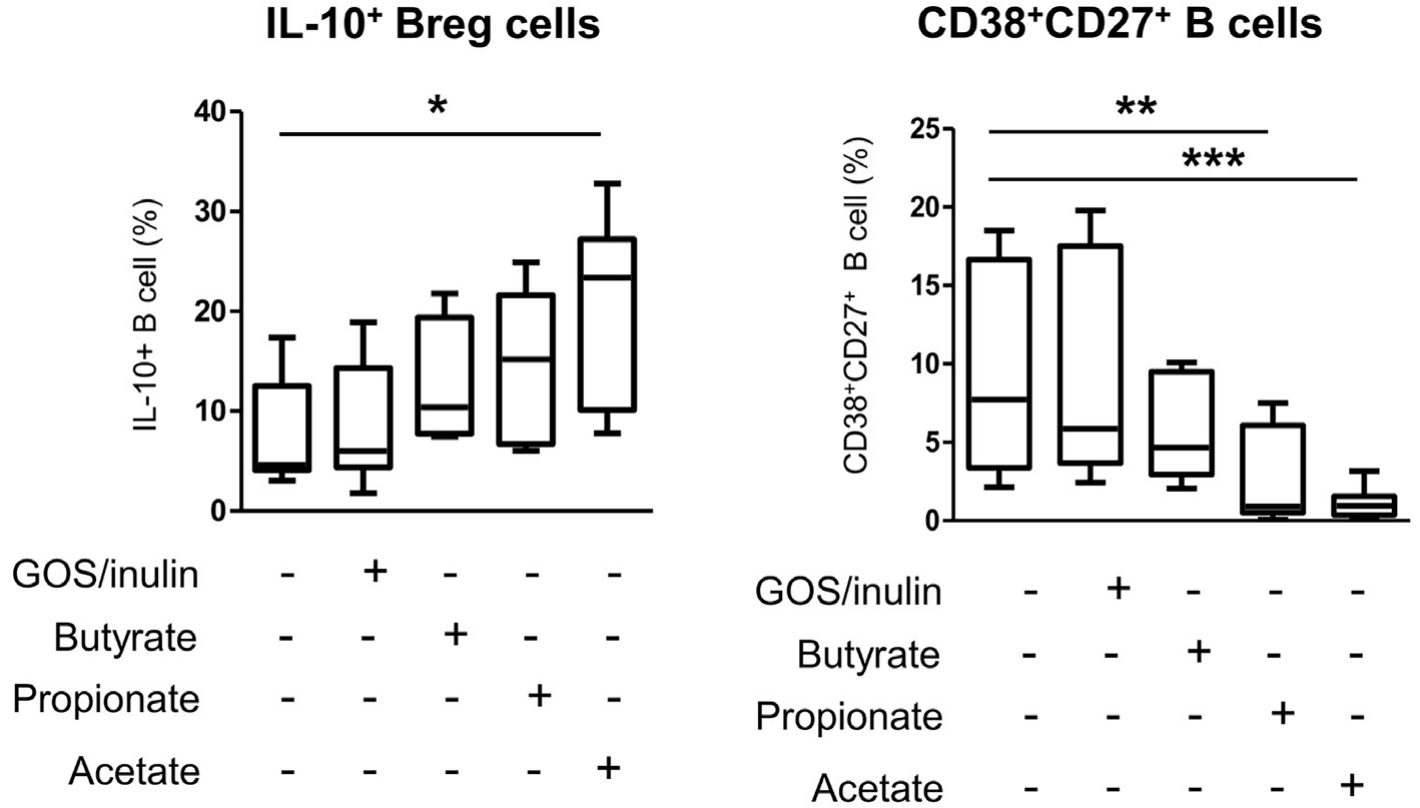
Effects of SCFA (butyrate, propionate and acetate) and GOS/inulin treatment *in vitro* on human IL-10^+^ Breg cell and CD27^+^CD38^high^ plasmablast frequencies. Human B cells isolated from PBMCs were cultured for 3 days with GOS/inulin, butyrate, propionate, acetate or no treatment as a control (n=11). The frequencies of IL-10^+^ B cells and plasmablast CD27^+^CD38^high^ B cells were estimated by flow cytometry (* = p<0.05, ** = p<0.01, *** = p<0.001).

We next investigated whether the increase in regulatory cells observed in mothers supplemented with prebiotics and their pups was due to a direct effect of prebiotics on B and T lymphocyte differentiation or an indirect effect due to SCFAs. The effect of SCFAs and prebiotics on cell differentiation was estimated *in vitro* using human PBMCs treated with GOS/inulin, butyrate, propionate or acetate. Only butyrate and propionate were associated with increased abundance of IL-10-secreting B cells compared to that in the control group (12.5% ± 2.4, 14.4% ± 2.9, 9% ± 2.3 and 8% ± 2 for butyrate, propionate, GOS/inulin and control, respectively, p>0.05) (**Figure 9**). In addition, acetate was associated with a significant increase in IL-10^+^ B cell rate (19.7% ± 3.6, p<0.05). In contrast, butyrate tended to decrease the frequency of CD27^+^CD38^+^ activated B cells compared to that of the control group (5.7% ± 0.96 and 9.2% ± 1.8 for butyrate and control, respectively, p>0.05), while GOS/inulin had no effect (9.1% ± 1.6, p>0.05). With regard to CD27^+^CD38^high^ B cells, propionate and acetate were associated with higher frequencies than the control condition (2.5% ± 0.7, p ≤ 0.01 and 1% ± 0.28, p ≤ 0.001, respectively). Surprisingly, SCFAs and GOS/inulin had no significant effect on CD9^+^ and CD25^+^ Breg differentiation *in vitro*, although acetate tended to increase CD9^+^ Breg cell frequency (**Supplementary Figure 1A, B**). Moreover, neither SCFAs nor GOS/inulin had a significant effect on Treg and DC differentiation *in vitro* (**Supplementary Figure C–E**). To summarize, acetate but not prebiotics decreases proinflammatory B cell differentiation for the benefit of anti-inflammatory IL-10-secreting Bregs.

## Discussion

This study highlights the impact of GOS/inulin supplementation during gestation on 1) the modulation of the mother’s gut microbiota, leading to increased SFCA production, 2) the transfer of metabolites such as acetate and amino acids in the amniotic fluid from mother to fetus, 3) the tolerogenic environment induced *in utero* in feto-maternal tissues, and 4) the establishment of tolerogenic and Breg-mediated immune imprinting in the fetus that remains associated with Tregs later in life.

Our study shows that GOS/inulin supplementation during gestation modifies the composition of the gut microbiota. In accordance with previous studies in mice (37, 38), the relative abundance of *Muribaculaceae* family members from the Bacteroidetes phylum was particularly high in the stool of prebiotic-supplemented mothers, and the *Lachnospiraceae* family was reshaped. These changes were not associated with a change in bacterial richness. In humans, dietary fiber intervention in healthy adults does not affect α-diversity despite changes in bacterial taxa (39). Interestingly, *Lachnospiraceae* and *Muribaculaceae* are two families in healthy subjects that are known as SCFA producers (40). Indeed, *Lachnospiraceae* were reported to be among the main producers of SCFAs (41), while *Muribaculaceae* abundance was strongly correlated with propionate production (42). In accordance with these findings, our results also highlight the effects of GOS/inulin supplementation on the functionality of the gut microbiota, showing increased concentrations of propionate, butyrate and particularly acetate in the stool of supplemented mothers. The increased concentration of SCFAs observed may be due to microbiota modification and/or increased substrate availability (43).

We also show here for the first time that prebiotic supplementation during gestation is associated with an increase in acetate levels in amniotic fluid. We hypothesize here that the acetate produced by dam gut microbiota is absorbed into the bloodstream and is transferred to the fetus by passing through the placental barrier or *via* the cord blood, ending up in the amniotic fluid. Indeed, fermentation performed by the intestinal microbiota is thought to be the primary source of exogenous acetate through uptake in the colon (44). Moreover, a correlation between cord blood and maternal blood acetate levels was previously described in humans, suggesting that maternal acetate may cross the placenta and therefore influence fetal acetate levels (24). However, it was also shown that acetate could originate from metabolic activity in tissues. Protein deacetylation and acetyl-CoA hydrolase activity also produce acetate but these contributions to the overall pool of acetate in the circulation was shown to be likely small (45). Interestingly, we also described increased concentrations of other metabolites in the amniotic fluid of prebiotic-supplemented dams, including factors related to amino acids, citric acid cycle, lipid metabolism, muscle metabolism, glycolysis and energy production. The pyruvate, end product of glycolysis, was found in higher concentration in the amniotic fluid of supplemented mothers with prebiotics and new evidence supports a pathway of *de novo* acetate production from pyruvate (46). In conclusion, the increase concentration of acetate is likely to come from the microbiota metabolic activity but may be also issued from the cell glycolysis. The other metabolites found in high concentration in the amniotic fluid are essential for fetal development since they possess potent bioactivity related to cellular growth and proliferation (47). Thus, we demonstrate here for the first time that prebiotic consumption during pregnancy may participate in the development of the fetus.

Interestingly, it was shown that SCFAs such as acetate exert immune regulatory effects by binding G-protein coupled receptors (GPRs), enhancing tolerogenic CD103^+^ DC and promoting Treg differentiation (9). Luu and colleagues also demonstrated that SCFA treatment *in vitro* increased the rate of IL-10^+^ B cells (48). Daien et al. showed that acetate promoted Breg differentiation both *in vivo* and *in vitro* (49). In the same way, we also demonstrated *in vitro* that acetate increases the abundance of human IL-10-secreting B cells. According to these observations, our results show that prebiotic supplementation during gestation is associated with an increased frequency of regulatory T and B cells at the feto-maternal interface, which may be due to the high level of circulating acetate. Indeed, a higher frequency of Tregs in the placenta and Bregs in both the placenta and the uterus was observed in prebiotic-supplemented dams, highlighting the establishment of a tolerant environment *in utero*. Acetate may be involved but other actors, especially cytokines (IL-10, TGFβ, IL-35), may be also implicated in the immune regulatory mechanism and this need to be further investigated. Recently, other key actors involved in human health were identified in the amniotic fluid: human milk oligosaccharides (HMO) (50). These sugars are able to act as prebiotics by inducing the growth of beneficial bacteria such as *Bifidobacterium* and *Lactobacillus* and acting directly on immune cells (51). We can hypothesis that GOS/Inulin supplementation could modified HMO profiles in the amniotic fluid. It would be interesting to test this hypothesis in the future. Finally, our results showed that prebiotic supplementation during pregnancy increased the rate of Breg cells in the fetus, and these cells associated with Treg cells were maintained later in life. We hypothesize that these Breg cells observed in the fetus are precursors of Treg cells observed in pups, as it was demonstrated that IL-10-secreting Breg cells drive naive T cell differentiation toward Tregs (52).

In conclusion, we show here for the first time the effect of a nutritional intervention during pregnancy on fetal outcomes, especially immunity. We have demonstrated that prebiotic supplementation during pregnancy fosters the establishment of a tolerogenic environment *in utero*, in the fetus and subsequently in the pup. We confirm here that pregnancy can be considered the very first “window of opportunity” for early-life education of the immune system. These insights have profound implications for human disease as they suggest that disease risk may begin at the earliest days of life, including the antenatal period. Our study is in accordance with the “DOHaD” concept, which links the state of health and risk of disease in later childhood and adult life with environmental conditions, especially food, in early life. An improved understanding of these events will therefore have great implications for the intervention and prevention of complex disease mechanisms. Modulation of the gut microbiota of pregnant women with nutritional strategies such as prebiotics therefore seems to be a promising strategy to prevent childhood diseases. Other nutritional strategies are currently under consideration [antioxidant, folate, vitamin D or probiotic supplementation (7) and the Mediterranean diet (53, 54)], especially for allergy prevention. In this context, we are currently running the PREGRALL clinical trial to determine whether antenatal prebiotic supplementation prevents atopic dermatitis in high-risk children (55).

## Data Availability Statement

The datasets presented in this study can be found in online repositories. The names of the repository/repositories and accession number(s) can be found below: NCBI SRA BioProject, accession no: PRJNA736212.

## Ethics Statement

Ethical review and approval was not required for the study on human participants in accordance with the local legislation and institutional requirements. The patients/participants provided their written informed consent to participate in this study. The animal study was reviewed and approved by Ethics Committee on Animal Experimentation of the Pays de la Loire region (CEEA.2011.52; 128; accreditation number: 14035).

## Author Contributions

Study conceptualization: CB, AS, and MB. Methodology; CB, AS, KM, DS, CC, SoB, and MB. Formal analysis: CB, AS, AD, BM-A, MC, and CC. Statistics: CB and VC. Resources: MB. Writing—original draft preparation: CB. Writing—review and editing: AS, SoB, CC, VC, DS, SeB, and MB. Supervision: MB. Project administration: MB. Funding acquisition: MB and SeB. All authors contributed to the article and approved the submitted version.

## Funding

This work was supported by the French government (Agence National pour la Recherche) and CHU de Nantes.

## Conflict of Interest

The authors declare that the research was conducted in the absence of any commercial or financial relationships that could be construed as a potential conflict of interest.

## Supplementary Material

Supplementary Figure 1 Effects of SCFA (butyrate, propionate and acetate) and GOS/inulin treatment *in vitro* on human Breg, Treg cell and Dc frequencies. Human B cells isolated from PBMCs were cultured for 3 days with GOS/inulin, butyrate, propionate, acetate or no treatment as a control (n=11). The frequencies of **(A)** CD9^+^ B cells, **(B)** CD25^+^ B cells, **(C)** CD4^+^CD25^+^Foxp3^+^ Treg cells, **(D)** pDCs and **(E)** cDCs were estimated by flow cytometry.
