# Supplementary figures and images for: Prebiotic Supplementation During Pregnancy Modifies the Gut Microbiota and Increases Metabolites in Amniotic Fluid, Driving a Tolerogenic Environment In Utero
Source: Front Immunol. 2021 Jul 14;12:712614. doi: 10.3389/fimmu.2021.712614 (PMC8317504; doi:10.3389/fimmu.2021.712614)

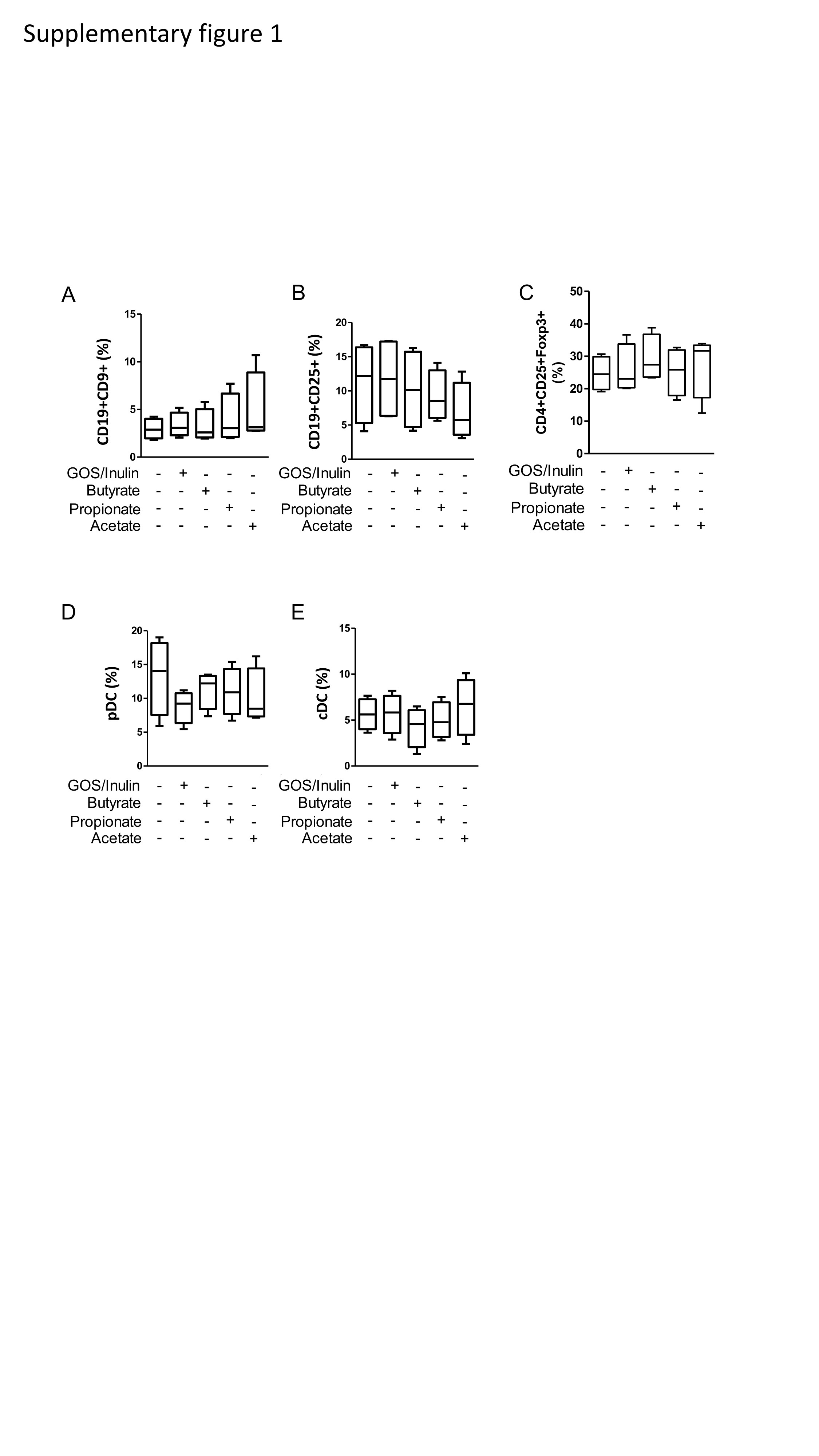

Supplement: Supplementary Figure 1 — Effects of SCFA (butyrate, propionate and acetate) and GOS/inulin treatment in vitro on human Breg, Treg cell and Dc frequencies. Human B cells isolated from PBMCs were cultured for 3 days with GOS/inulin, butyrate, propionate, acetate or no treatment as a control (n=11). The frequencies of (A) CD9+ B cells, (B) CD25+ B cells, (C) CD4+CD25+Foxp3+ Treg cells, (D) pDCs and (E) cDCs were estimated by flow cytometry. [file Image_1.jpeg]
